# Network analysis of influencing factors on rural residents’ health communication behaviors: a social-ecological perspective

**DOI:** 10.3389/fpubh.2026.1825320

**Published:** 2026-06-08

**Authors:** Yahong Guo, Xinjin Li, Cailing Yang, Yanhua Ning, Lingna Liu, Jing Shi, Weijuan Kong

**Affiliations:** 1 General Hospital of Ningxia Medical University, Yinchuan, China; 2 School of Nursing, Ningxia Medical University, Yinchuan, China

**Keywords:** health communication, influencing factors, network analysis, rural residents, social-ecological model

## Abstract

**Objective:**

This study aims to construct a network structure of influencing factors on rural residents’ health communication behaviors based on the Social-Ecological Model, employing network analysis techniques to identify core factors. The findings will serve as a theoretical and practical reference for developing evidence-based health promotion programs tailored to rural areas.

**Methods:**

This study was a cross-sectional survey. A total of 1,549 rural residents from 10 villages of Ningxia Province in China were recruited between July-20th and September-20th, 2023. Based on the Social-Ecological Model of rural residents, three levels of factors: individual-level, interpersonal-level, and environmental-level were assessed using questionnaire. Multi-level regression models were employed using SPSS. In addition, network analysis of factors influencing health communication behaviors of rural residents was conducted using R4.4.2 software.

**Results:**

The mean score of health communication behaviors of rural residents was (5.16 ± 1.68). Multiple-level regression results showed that seven individual level factors (gender (*β* = 0.19, *p* < 0.01), age (*β* = 0.10, *p* < 0.01), occupation (*β* = −0.07, *p* < 0.01), income (*β* = 0.12, *p* < 0.001), activities of daily living (*β* = −0.05, *p* < 0.05), perceived ease of use (*β* = 0.21, *p* < 0.001), and media involvement (*β* = 0.21, *p* < 0.001)), three interpersonal level factors (types of chronic illnesses in family members (*β* = 0.17, *p* < 0.001), family members’ medical background (*β* = −0.05, *p* < 0.05), and social support (*β* = 0.07, *p* < 0.001)), and 1 environmental level factor (the frequency of health information dissemination by village committees) influenced the level of health communication behaviors among rural residents. The network analysis revealed that social support exhibited the most prominent strength, closeness, and betweenness, with interactions occurring across hierarchical levels.

**Conclusion:**

The overall level of health communication behaviors among rural residents remains low, which is shaped by multi-level (individual, interpersonal, and environmental) and multi-factorial interactions. Notably, social support is a central factor in this complex network, serving as both a pivotal hub and a mediating mechanism connecting the various variables. Future research should adopt a multi-dimensional approach to enhance health communication behaviors through coordinated interventions targeting individual agency, interpersonal networks, and environmental policies.

## Introduction

Health Communication is defined as a structured process involving the formulation, dissemination, circulation, and shared understanding of health-related information through diversified channels and modalities, aiming to safeguard and enhance health status of human beings, and is the specific practice and further deepening of health information communication behavior in the medical field (1). The primary goal of health communication is to increase public awareness of health risks and reinforce positive health actions, thereby changing the attitudes and behaviors of individuals or groups (2). Health communication, as a crucial method for enhancing the health literacy of the public and tackling human health issues, can impact health status by broadening individuals’ health knowledge and modifying their health behaviors (3, 4).

According to the seventh national census, China’s rural population reached 590 million, accounting for 36.11% of the national population (5). Although the proportion of China’s rural population has continued to decline over the past decade, the overall number is still sizable. In recent years, with economic development and the implementation of health-related policies such as the new healthcare medical reform, the health status of rural residents in China has improved to a greater extent (6, 7), but rural residents still retain many unhealthy habits and misconceptions, which impede the improvement of their overall health (8, 9). Under the current social-economic environment of China, conducting health communication activities and advocating healthy lifestyles are the most economical and effective ways to improve people’s health literacy (10).

Kshatri et al. (11) found that good interpersonal communication is the main source of health information for rural residents. Gretchen et al. (12) found in their survey that health behaviors of residents improve after health knowledge dissemination related to the global issue of food safety was carried out in rural areas. Domestic work on health communication in rural areas has been conducted since the 1990s, mostly focusing on the communication phenomenon in a few provinces and cities, mainly focusing on the publicity and education of health knowledge, or analyzing the constraints and needs of health communication based on cases and proposing countermeasures (13, 14). Although the research on health communication in rural areas by relevant scholars has been on the rise in recent years, the research on influencing factors has mostly focused on general demographic characteristics or a certain type of single factor, and lacks a comprehensive analysis of the influencing factors of health communication among rural residents (15). It is widely accepted that behavior is the result of a combination of individual and environmental factors (16), so studies that focus only on certain isolated factors tend to produce biased results by failing to consider the effects of other factors or their interactions with the dependent variables. From a behavioral perspective, individual behavior is determined by a variety of factors that interact with each other in complex ways, sometimes superimposing and reinforcing each other, sometimes counteracting and weakening each other. Therefore, it is necessary to conduct multi-level and multi-dimensional empirical studies on the factors related to health communication behaviors of rural residents under the social-ecological framework (17, 18).

The social-ecological theoretical model emphasizes that behaviors are not solely the result of individual choices, but are shaped by the interaction of complex factors at multiple levels ranging from personal characteristics to broader environmental and social influences (17). The comprehensive systems study based on the social-ecological framework has methodological advantages that overcome the bias of results from fragmented studies of other theories (17). From previous studies, the factors related to health communication behaviors of rural residents based on the social-ecological model include four levels: individual, interpersonal, environmental, and policy, and the empirical studies mainly focused on the individual, interpersonal, and environmental levels (19–21). Among them, the individual level focuses on social-demographic characteristics (19), the interpersonal level focuses on family and peer support, and the environmental level focuses on factors in the communication environment that are closely related to daily life (20, 21). Although there are some studies in European and the United States that have used a social-ecological approach to explore influencing factors (22–24), the findings are not necessarily applicable to China due to differences in social-economic and cultural backgrounds. Currently, there is limited study based on social-ecological models to determine the influencing factors of health communication behaviors of Chinese rural residents.

Recent years have witnessed a growing interest in applying network analysis to understand health behaviors in rural populations. or instance, Yang et al. (25) utilized network analysis to map the complex interrelationships among health-related behaviors and chronic non-communicable diseases, revealing how specific behavioral factors occupy central positions in influencing overall health outcomes in Chinese communities. Similarly, Li et al. (26) employed a network perspective to explore the prevalence of depression and anxiety, identifying key nodes and bridge symptoms within the psychological distress network among patients with chronic diseases. These studies underscore the utility of network approaches in visualizing relational data beyond traditional regression models. However, a notable gap remains regarding the application of network analysis to health communication behaviors specifically within the rural context, particularly through a multi-level theoretical lens.

**Figure 1:**
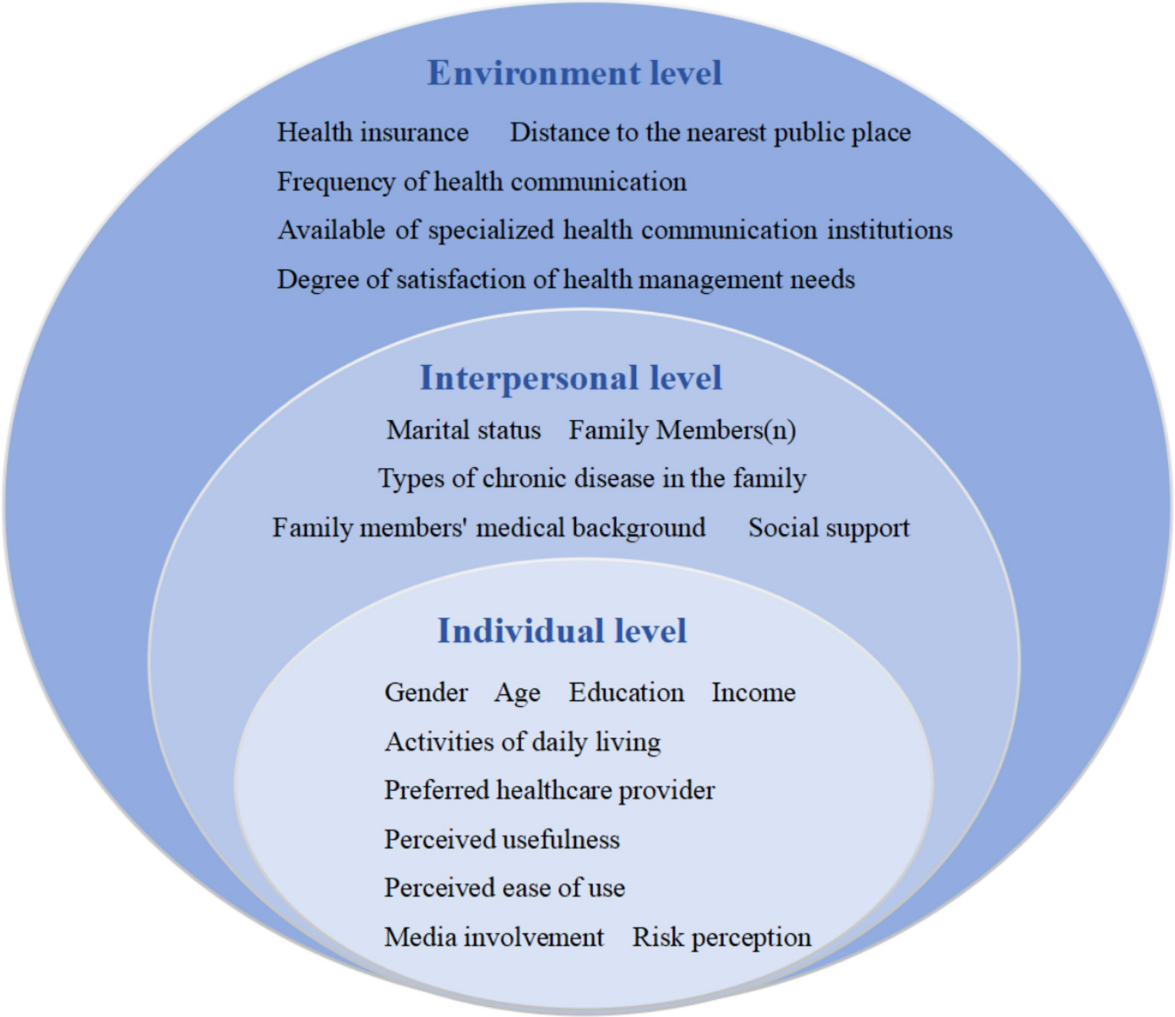
work diagram of the social-ecological factors of health communication behaviors in rural residents.

Therefore, the purpose of this study was to investigate the social-ecological correlates of health communication behaviors in Chinese rural residents and to identify the core influencing factors by combining network analysis. Specifically, the study aimed to explore the individual-level factors (characteristics, chronic diseases, activities of daily living, preferred healthcare provider, perceived usefulness, perceived ease of use, media involvement, risk perception), interpersonal-level factors (marital status, family members(n), types of chronic disease in the family, family members’ medical background, social support), and environment-level factors (health insurance, distance to the nearest public place, frequency of health communication in the village, availability of specialized health communication institutions in the village, and degree of satisfaction of health management needs) were associated with each other (Figure 1). The findings of the study will provide empirical evidence for the promotion of health communication in rural China and the development of related policies.

## Methods

### Participants and data collection

**Figure 2:**
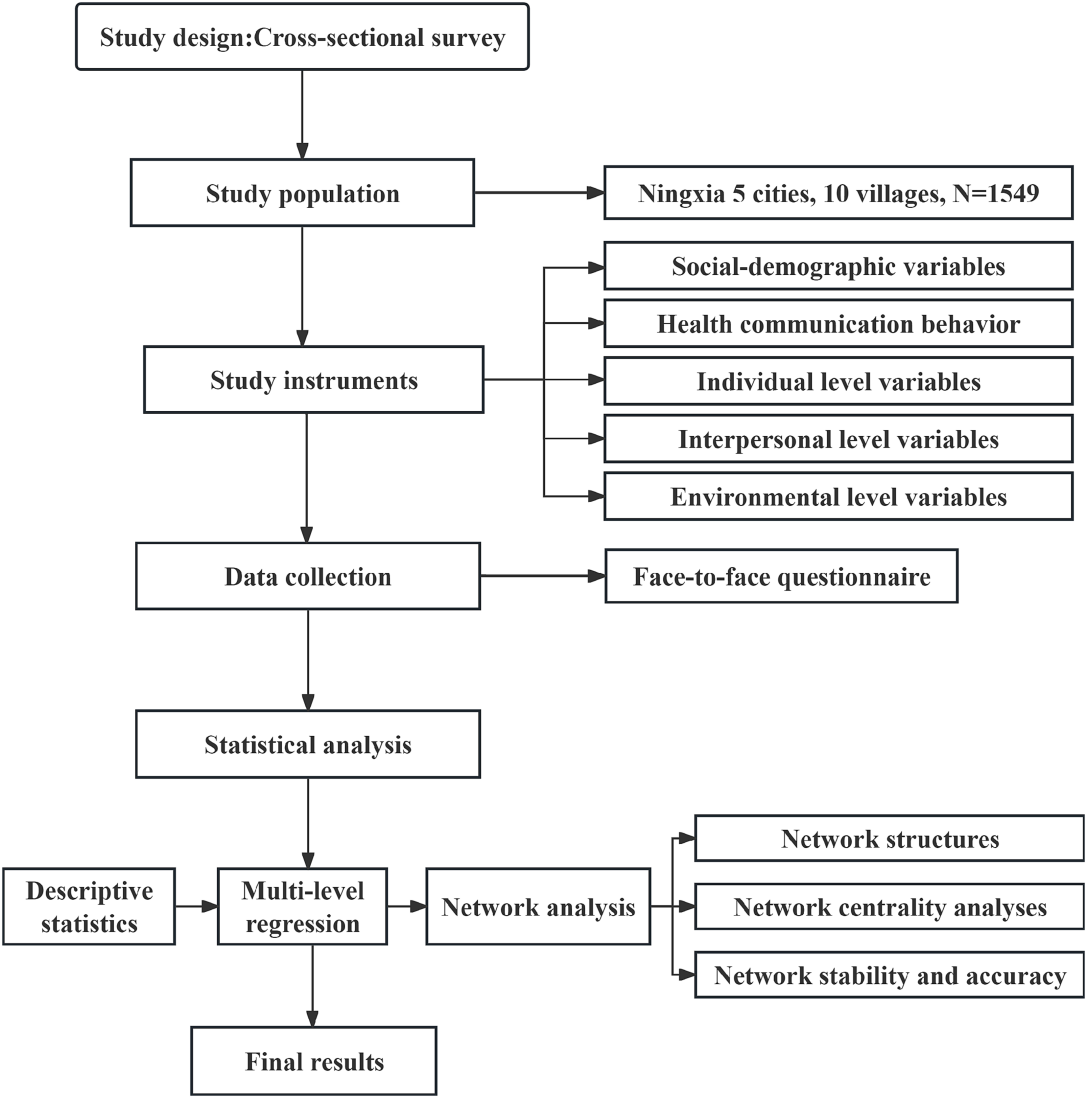
Flow diagram of the overall methodology.

The data used for the study came from a cross-sectional design, population-based survey conducted from July to September 2023. The whole cluster sampling method was used to select one county in each of the five cities of Ningxia and two administrative villages in each county as sample villages, and questionnaires were administered to all permanent residents within the jurisdiction of the 10 sample villages. The flow diagram of the overall study methodology is shown in Figure 2.

To ensure the robustness of the statistical analysis, a power analysis was conducted beforehand to calculate how many participants would be needed. The minimum sample size for this study was determined by multivariate logistic regression: the required sample size should be at least 10 times or more than the number of independent variables (27). With 21 independent variables and 80% statistical power, 252 participants were required. According to the principle that the sample size of the structural equation model should be based on 200 at least 10 additional participants for each additional variable (28), a non-response rate of 10 to 15% was estimated, requiring at least 472 participants.

Participants were individuals aged 18 years or older in 10 villages in Ningxia Province, China, who were able to communicate briefly and agreed to participate in the study. They must have a Ningxia household registration or have lived in a rural area for at least 12 months. However, rural residents with severe physical illness and psychological or psychiatric conditions were excluded. All eligible participants were invited to participate in a face-to-face survey, which typically lasted 10–15 min. Informed consent was obtained from each participant before conducting the survey. The study protocol was approved by the Ethics Review Committee of Ningxia Medical University (ID: 2023-G179).

### Instruments

#### Social-demographic variables

The survey included questions on social-demographic factors including age, gender, marital status, education level, employment status, and income/month (in Chinese Yuan, CNY).

#### Health communication behaviors

Health communication behaviors (29) were assisted through three questions: “the average number of times per month you actively seek health information”, “the average number of times per month you receive health information from different sources”, and “the average number of times per month you actively forward or share health information”. The three items were scored on a 5-point Likert scale ranging from 5 (“more than 15 times”), 4 (“10–15 times”), 3 (“6–10 times”), 4 (“1–5 times”) and 1 (“0 times”). The reliability and validity have been supported in the Chinese population (27) and the internal consistency of this study was acceptable(*α* = 0.715).

#### Individual level variables

Demographic information about rural residents, including gender, age, education, income/month, chronic disease, activities of daily living and preferred healthcare providers, was collected through a questionnaire. The Perceived Usefulness Scale (4 items), Perceived Ease of Use Scale (3 items), Media Involvement (3 items) and Perceive Risk Scale (3 items) were adapted from previously validated scales (30–32). The example of items in the perceived usefulness was “Health information access and sharing has helped me and others increase our health knowledge.” The example of items in the perceived ease of use was “Health information is easy to share, disseminate and interact with.” The example of items in the media involvement was “I am able to recognize, understand and determine whether health information is useful or not.” The example of items in the risk perception was “Impaired health has a negative impact on life.” A 5-point Likert scale was used to measure the level of agreement with the given items (1 = strongly disagree, 5 = strongly agree). The internal consistency of subscales in this study were 0.951, 0.861, 0.859 and 0.953, respectively.

#### Interpersonal level variables

Marital status, family members (n), type of chronic disease in the family, and family members’ medical background of rural residents were collected through a self-reported questionnaire. Additionally, the social support of rural residents was assessed using the Social Support Rating Scale (SSRS) developed by Xiao (33). The scale consists of 10 items in 3 dimensions: objective support, subjective support, and support utilization. The scale was scored on a 5-point Likert scale, with a score of less than 33 as low social support, 33–45 as average social support, and greater than 45 as high social support, with higher scores indicating higher levels of social support (Cronbach’s *α* = 0.896).

#### Environmental level variables

Through a literature review (6, 7, 15), the environmental dimension consisted of five items including “the type of health insurance you currently hold” “the distance(in kilometers) from your home to the nearest public recreational facility”, “Frequency of health communication in the village in the past year”, and “Availability of specialized health communication institutions in the village (e.g., health promotion associations)”. “Whether the current National Basic Health Service Standards adequately meets your health management needs”. Participants could answer truthfully according to their actual situation.

### Statistical analyses

Microsoft Excel 2019 software was used to establish the database, SPSS 26.0 software was used for statistics and description of data, and R4.4.2 software was used for network analysis. Descriptive statistics were calculated, presenting continuous variables as mean ± standard deviation (M ± SD) and discrete variables as frequency (%). To examine the association between social-ecological factors and health communication behaviors, multiple-level regression models were employed. The significance level for all analyses was set at *p* < 0.05 (two-tailed).

Following established practices in network psychometrics (34), only variables that demonstrated statistically significant associations with health communication behaviors in the multilevel regression models were retained for network analysis. This approach aims to avoid overfitting and improve the stability and interpretability of the network structure. Firstly, Gaussian graphical model (GGM) was used in this study because it can effectively identify the unique correlation between variables after controlling for confounders and has been widely used in cross-sectional data containing continuous and ordinal variables (35), and the network was constructed by using the EBICglasso function in the qgraph package, the nodes in the network represented the observed variables, and the connecting lines between two nodes became the edges, and the coarser edges indicated the stronger associations between the nodes; secondly, the centrality metrics of the network nodes are computed, which are mainly measured through betweenness, closeness and strength. Strength is the main index of centrality, which is the sum of the weighted values of a node’s connections to other nodes, reflecting the importance of nodes in the network; closeness is the sum of the distances from a node to all other nodes, reflecting the closeness of a node’s connections to other nodes; betweenness is the number of times a node is passed by how many nodes with the shortest distances between them, reflecting the node’s importance in the connections of other nodes (36, 37). Stability was quantified by calculating the correlation stability (CS) coefficient which is the maximum proportion of cases that can be removed from the sample to retain, with 95% certainty, a correlation of at least 0.7 between the original centrality indices (full sample) and those derived from subsamples. It is recommended that CS coefficient >0.25 is regarded as a minimum threshold for index stability, while indices with CS coefficient>0.5 are considered as sufficiently stable (38). The accuracy of the network edge weights was assessed by calculating the 95% CI of the edge weights through 1,000 nonparticipating bootstrap sampling, with narrower CIs indicating more reliable accuracy of the network structure edge weights (39).

## Results

### Sample characteristics

A total of 1,549 individuals were recruited to participate in the study, and the questionnaire response rate was 96.93%. Among the participants, 922 (59.5%) were women. The highest proportion of participants was older adults (43.6%). The participants had a relatively low educational level (68.6% had primary or lower education level and 3.3% had undergraduate or higher education level). More than three-quarters of the participants were married (85.1%). 1,011 participants had more than four members in their families (65.3%). Most rural residents do not have a medical background of family (85.9%). Many rural residents have low economic conditions, with only 44 (2.8%) of them earning more than 5,000 CNY/month. Only a very small number of rural residents do not have health insurance (1.9%). 32.1% of rural residents reported suffering from more than two diseases. More details of the participants characteristics can be found in Table 1.

**Table 1:** Sample studied variables (*N* = 1,549).

Variables		Mean ± SD or frequency (percentage)
Individual level variables		
Gender	Male	627 (40.5)
	Female	922 (59.5)
Age(year)	18–29	136 (8.8)
	30–39	160 (10.3)
	40–49	208 (13.4)
	50–59	370 (23.9)
	≥60	675 (43.6)
Education	Primary school or lower	1,062 (68.6)
	Middle school	303 (19.6)
	Senior middle school	88 (5.7)
	College	45 (2.9)
	Undergraduate or higher	51 (3.3)
Occupation	Farmer	837 (54.0)
	Migrant workers	133 (8.6)
	Staff of government agencies, enterprises/public institutions	48 (3.1)
	Student	59 (3.8)
	Unemployed	369 (23.8)
	Business/Service workers	63 (4.1)
	Others	40 (2.6)
Income/month (in CNY)	<1,000	706 (45.6)
	1,000–1999	395 (25.5)
	2000–2,999	240 (15.5)
	3,000–3,999	124 (8.0)
	4,000–4,999	40 (2.6)
	≥5,000	44 (2.8)
Activities of daily living	Able to take care of themselves completely	1,414 (91.3)
	Some are self-reliant	135 (8.7)
Preferred healthcare provider	Village clinic	447 (28.9)
	Community health service organizations	283 (18.3)
	County hospitals	216 (13.9)
	Municipal hospitals	300 (19.4)
	Nearby clinics	175 (11.3)
	Others	128 (8.3)
Individual level variables		
Perceived usefulness		15.70 ± 3.74
Perceived ease of use		9.75 ± 3.44
Media involvement		10.02 ± 3.30
Risk perception		13.15 ± 2.67
Interpersonal level variables		
Marital status	Single	97 (6.3)
	Married	1,318 (85.1)
	Widowed	134 (8.6)
Family members(*n*)	1	65 (4.2)
	2	323 (20.9)
	3	150 (9.7)
	≥4	1,011 (65.3)
Types of chronic disease in the family	0	617 (39.8)
	1	435 (28.1)
	≥ 2	497 (32.1)
Family members’ medical background	Yes	218 (14.1)
	No	1,331 (85.9)
Social support	33.34 ± 5.25	
Environmental level variable		
Health insurance	No	29 (1.9)
	Basic medical insurance for employees	52 (3.3)
	Basic medical insurance for urban and rural residents	1,468 (14.5)
Distance to nearest public place(kilometer)	<1	1,193 (5.7)
	1~	224 (77.0)
	2~	88 (94.8)
	3~	12 (0.8)
	4~	32 (2.1)
Availability of specialized health communication institutions in the village	Yes	445 (28.7)
	No	1,104 (71.3)
Degree of satisfaction of health management needs	Satisfy	1,388 (89.6)
	Not satisfied	161 (10.4)
Dependent variable		
Health Communication behaviors		5.16 ± 1.68

### Multiple-level regression model of health communication behaviors

For health communication behaviors, the multi-level regression model showed that 7 out of 11 individual-level factors significantly predicted health communication behaviors (*R^2^* = 0.182, *p* < 0.001; see Table 2, Model 1), including gender(*β* = 0.19, *p* < 0.01), age (*β* = 0.10, *p* < 0.01), occupation (*β* = −0.07, *p* < 0.01), income/month (*β* = 0.12, *p* < 0.001), activities of daily living (*β* = −0.05, *p* < 0.05), perceived ease of use (*β* = 0.21, *p* < 0.001), and media involvement (*β* = 0.21, *p* < 0.001). Among interpersonal-level, types of chronic disease in the family (*β* = 0.17, *p* < 0.001), family members’ medical background (*β* = −0.05, *p* < 0.05) and social support (*β* = 0.07, *p* < 0.001) positively predicted health communication behaviors after controlling for individual factors (*R^2^* = 0.212, *p* < 0.001; see Table 2, Model 2). In addition, after controlling for interpersonal and individual factors, frequency of health communication in the village(year) (*β* = 0.03, *p* < 0.05) significantly positively predicted health communication behaviors (*R^2^* = 0.216, *p* < 0.001; see Table 2, Model 3). With each addition of a dimension factor in the social-ecological model, the fit of the regression model continues to increase.

**Table 2:** Multi-level linear regression predicting rural residents’s health communication behavior from social-ecological correlates (*N* = 1,549).

Variable	Model 1		Model 2		Model 3	
	B (95%CI)	β	B (95%CI)	β	B (95%CI)	β
Individual level variables						
Gender	0.24 (0.16 ~ 0.31)	0.19^***^	0.12 (0.04 ~ 0.21)	0.10^**^	0.12 (0.04 ~ 0.21)	0.10^**^
Age (year)	0.33 (0.17 ~ 0.48)	0.10^***^	0.25 (0.09 ~ 0.40)	0.07 ^***^	0.24 (0.08 ~ 0.40)	0.07^***^
Education	0.02(−0.09 ~ 0.12)	0.01	0.04(−0.07 ~ 0.14)	0.02	0.02(−0.09 ~ 0.13)	0.01
Occupation	−0.06(−0.10 ~ −0.02)	−0.07^**^	−0.05(−0.09 ~ −0.01)	−0.06^**^	−0.05(−0.09 ~ −0.01)	−0.06^*^
Income/month (CNY)	0.16 (0.10 ~ 0.23)	0.12^***^	0.16 (0.10 ~ 0.23)	0.12^***^	0.16 (0.09 ~ 0.23)	0.12^***^
Activities of daily living	−0.27(−0.53 ~ −0.01)	−0.05^*^	−0.35(−0.6 ~ −0.09)	−0.06^**^	−0.37(−0.63 ~ −0.11)	−0.07^**^
Preferred healthcare provider	0.04 (0.00 ~ 0.09)	0.04	0.04(−0.01 ~ 0.08)	0.03	0.04(−0.01 ~ 0.08)	0.04
Perceived usefulness	0.02(−0.01 ~ 0.04)	0.04	0.01(−0.02 ~ 0.03)	0.02	0.00(−0.02 ~ 0.03)	0.01
Perceived ease of use	0.10 (0.06 ~ 0.14)	0.21^***^	0.10 (0.06 ~ 0.14)	0.20^***^	0.10 (0.06 ~ 0.13)	0.20^***^
Media involvement	0.11 (0.07 ~ 0.15)	0.21^***^	0.11 (0.07 ~ 0.15)	0.21^***^	0.11 (0.07 ~ 0.15)	0.21^***^
Risk perception	−0.01(−0.04 ~ 0.02)	−0.01	−0.01(−0.04 ~ 0.02)	−0.01	−0.01(−0.04 ~ 0.02)	−0.01
Interpersonal level variables						
Marital status			0.18(−0.04 ~ 0.40)	0.04	0.18(−0.04 ~ 0.39)	0.04
Family members(*n*)			0.06(−0.03 ~ 0.14)	0.03	0.06(−0.03 ~ 0.14)	0.03
Types of chronic disease in the family			0.33 (0.23 ~ 0.43)	0.17^***^	0.33 (0.23 ~ 0.43)	0.17^***^
Family members’ medical background			−0.23(−0.45 ~ −0.01)	−0.05^*^	−0.22(−0.44 ~ −0.01)	−0.05^*^
Social support			0.02 (0.01 ~ 0.04)	0.07^***^	0.02 (0.01 ~ 0.04)	0.07^***^
Environmental level variables						
Health insurance					−0.12(−0.36 ~ 0.12)	−0.02
Distance to nearest public place (kilometer)					0.06(−0.04 ~ 0.15)	0.03
Frequency of health communication in the village					0.07(−0.04 ~ 0.18)	0.03^*^
Availability of specialized health communication institutions in the village					0.07(−0.11 ~ 0.24)	0.02
Degree of satisfaction of health management needs					−0.23(−0.48 ~ 0.02)	−0.04
*R^2^*	0.182		0.212		0.216	
*∆R^2^*	0.176		0.203		0.205	
*F*	31.129^***^		25.697^***^		19.119^***^	

*P < 0.05; **P < 0.01; ***P < 0.001.

### Network analysis of factors influencing health communication behaviors

Based on the results of multiple-level logistic regression analysis, this study included statistically significant factors related to health communication behaviors in the network analysis in order to explore the interactions between factors related to residents’ health communication behaviors, and then to filter out the most influential factors.

#### Network structures

**Figure 3:**
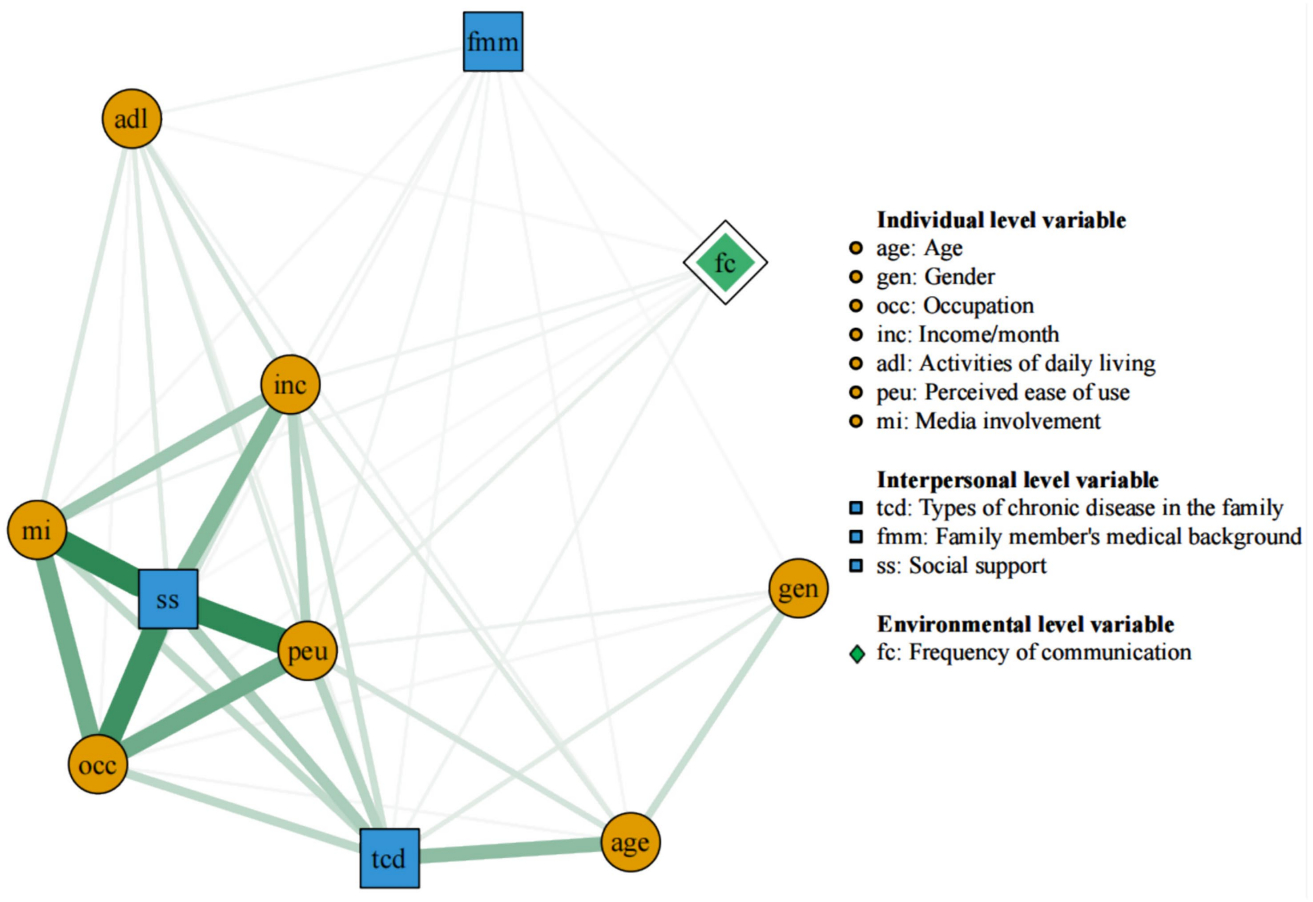
Network structure between factors of health communication behaviors of rural residents.

The complete network consists of 11 nodes and 55 edges, 47 of which have non-zero weights, as shown in Figure 3. The orange nodes in the figure indicate 7 variables at the individual level, the blue nodes indicate 3 variables at the interpersonal level, and the red nodes indicate 1 variable at the environmental level. Connecting edges with strong correlations include: social support, perceived usefulness, media involvement, age, occupation, income/month, and types of chronic disease in the family with strong correlations.

#### Network centrality analyses

**Figure 4:**
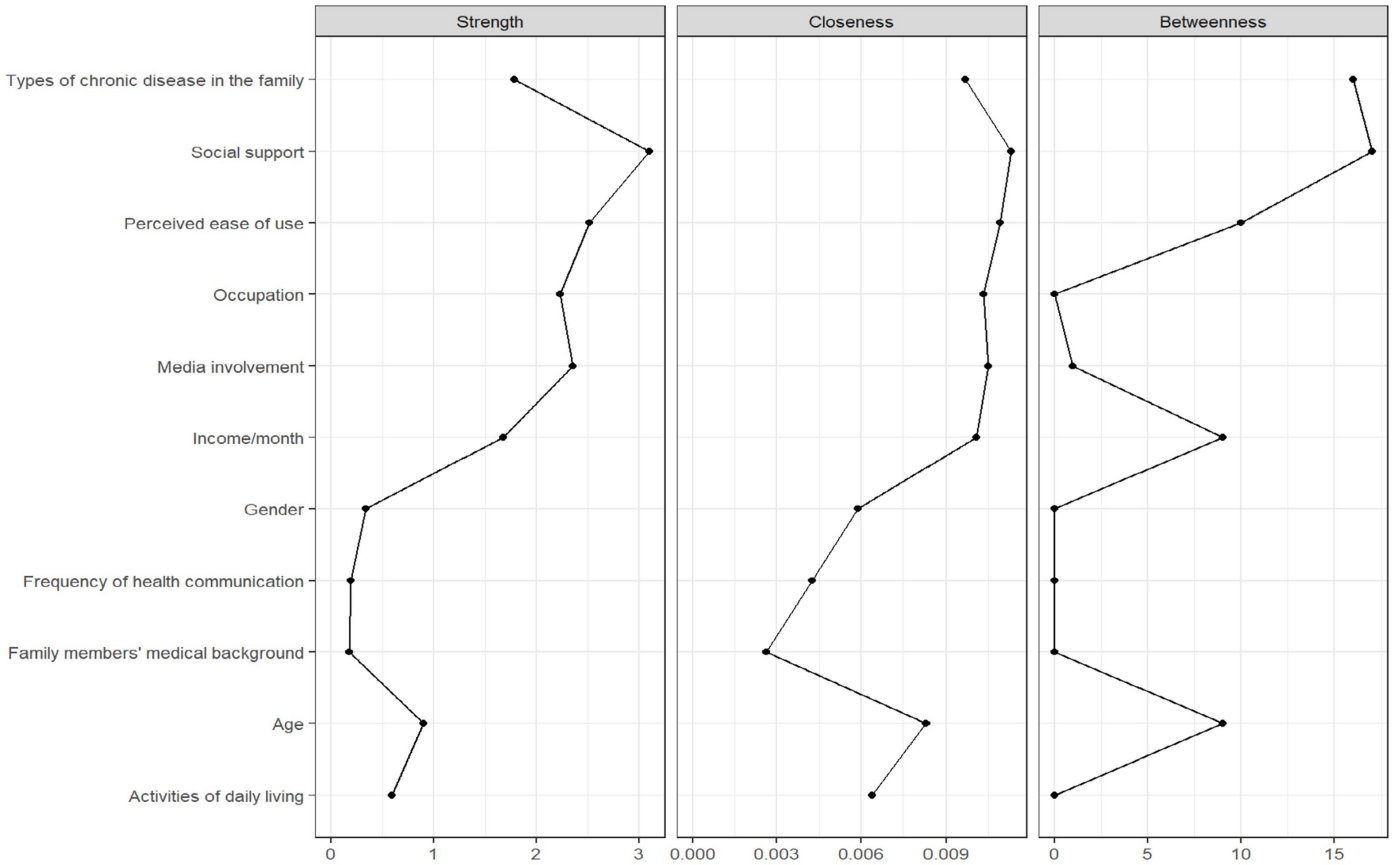
Network node centrality indicators of factors influencing health communication behaviors of rural residents.

The results of the network centrality index analysis showed that the node with the highest centrality index in the network model was social support, highlighting its centrality in the network, as shown in Figure 4. Hierarchical comparison revealed comparable strength centrality between individual and interpersonal levels, with the interpersonal level showing the highest betweenness centrality. The environmental level variable had relatively lower centrality (Table 3).

**Table 3:** Summary of centrality metrics across hierarchical levels.

Variable level	Nodes	Strength (M ± SD)	Closeness (M ± SD)	Betweenness (M ± SD)
Individual level	7	0.30 ± 0.11	0.81 ± 0.11	0.43 ± 0.39
Interpersonal level	3	0.30 ± 0.16	0.81 ± 0.17	0.45 ± 0.56
Environmental level	1	0.21	0.71	0.08

No standard deviation is reported for the environmental level due to a single node.

#### Network stability and accuracy

**Figure 5:**
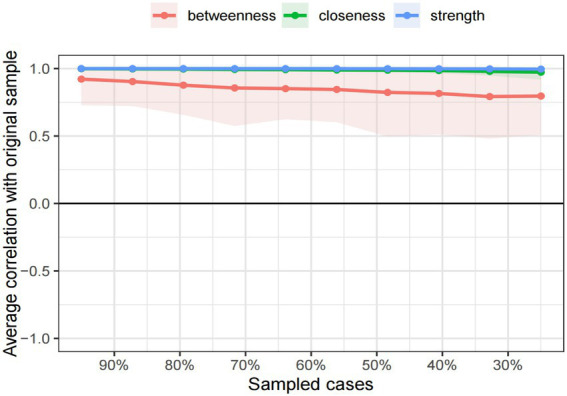
Network model center indicator stability assessment.

**Figure 6:**
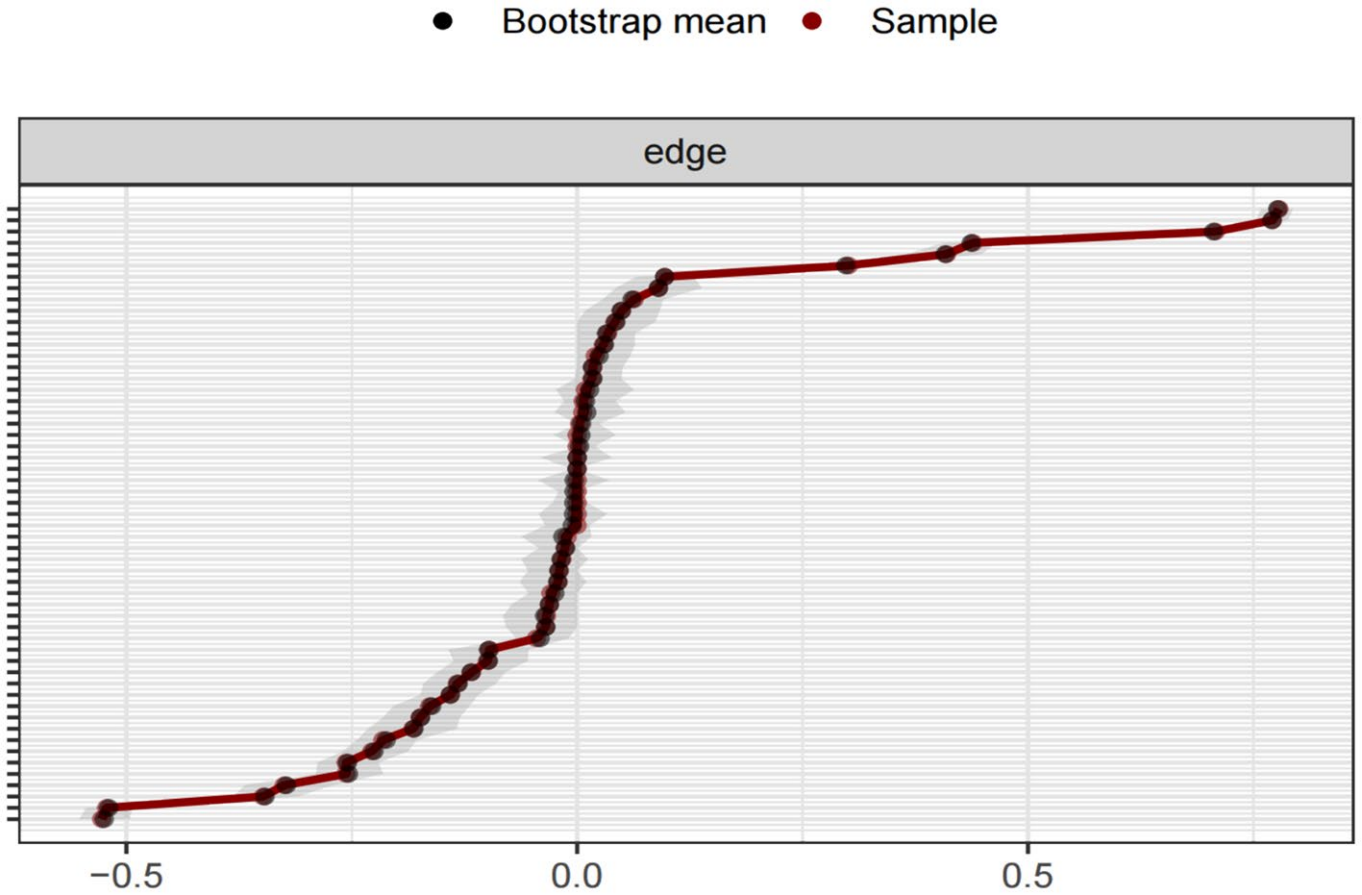
Network edge stability evaluation.

The stability estimation results of the network centrality index showed that the CS values of strength, betweenness and closeness are all greater than 0.7, indicating that the network has high stability, see Figure 5. The bootstrap results of the edge weights of the network are shown in Figure 6, with the black line as the bootstrap mean value of the connecting edge weights, the red line as the original sample value of the connecting edge weights, and the grey area as the 95% confidence interval of the bootstrap results. From the figure, it can be seen that the confidence intervals of the edge weights are narrower and close to the original sample results, which indicates that the network structure is accurate and reliable.

## Discussion

This study provides novel insights into rural residents’ health communication behaviors from a social-ecological network perspective. First, previous studies have mostly focused on single-level or demographic factors, while few have systematically revealed the interactive relationships across individual, interpersonal, and environmental levels. Second, this study identifies social support as a core hub factor that bridges different ecological levels, which offers a clear and actionable intervention target for improving health communication in rural areas. Collectively, these findings extend current understanding of how multilevel factors interact to shape health communication and provide new evidence for developing targeted health promotion strategies in rural China (Table 3).

### The level of health communication behaviors among rural residents is relatively low

The mean health communication behaviors score of rural residents was (5.16 ± 1.68), which was low compared to the median score of 9, similar to the results of previous studies (40). Residents in rural areas mainly rely on traditional health communication methods, which are characterised by top-down unidirectional information dissemination and often lack effective audience interaction and participation, affecting the utilisation of health information. Concurrently, the mismatch between communicated content and audience needs further undermines the effectiveness of communication and makes it difficult to develop sustainable health awareness or behavior changing among rural populations (41). The Social-Ecological Model emphasises that analysing individual factors in isolation does not adequately reflect the complexity of health-related behaviors; instead, an integrative framework that accounts for individual, interpersonal, and environmental factors can provide comprehensive insights into the determinants of behavior (42). This systematic perspective calls for multisectoral interventions to promote the health of rural populations through coordinated strategies that span individual capacities, social networks, and community resources.

### Individual, interpersonal and environmental factors influencing health communication behaviors of rural residents

Individual-level factors reveal significant differences in the way rural residents of different age groups access, comprehend, and apply health information. Compared to younger people, older people are less likely to use new media platforms such as social media or the internet, relying instead on traditional media such as television, radio, or interpersonal communication (43). These traditional channels may not provide sufficiently rich or timely health information. Furthermore, age-related declines in cognitive and memory abilities can affect older adults’ comprehension and retention of health information, making it difficult for them to process large amounts of data quickly or apply this knowledge effectively in their daily lives (44). Health communication research inherently possesses a strong practical orientation. Women, often positioned as the emotional hub of the family unit, are socially expected to fulfil caregiving responsibilities and household chores, thereby occupying a pivotal role in family-based health communication (45). A report by Susanah Fox and Lee Rainie at the Pew Research Center found that women not only search for health information online more frequently than men, but also show greater diversity in the type and content of health topics they pursue (46). Factors such as career stability and income level (47) contribute to differences in health investment behavior. People with stable jobs and higher incomes usually enjoy better access to healthcare service and information resources, and usually prioritise personal health investments, such as purchasing premium healthcare products, attending private seminars, or consulting healthcare professionals. Conversely, low-income groups face economic constraints that limit their ability to allocate resources to health-related needs.

Activities of daily living refer to an individual’s ability to perform basic daily tasks independently (48). For rural residents with less ability to perform ADLs, they face greater health challenges such as limited mobility and difficulty in accessing information, which may limit their participation in health communication activities. Additionally, media involvement and perceived ease of use were found to be positive predictors of health communication behavior among rural residents, which is consistent with the findings by Wang et al. (49). This suggests that after receiving health information, rural residents will consciously or subconsciously assess the accessibility, professionalism, and usefulness of the information. Information that is easily accessible, professionally sourced, and practical is more likely to elicit their desires to share, thereby promoting further participation in health communication content.

At the interpersonal level, people with chronic diseases scored significantly higher on health communication behaviors than people without chronic conditions (50). This suggests that when people perceive themselves to be in poorer health than their peers-being sick more often or having a history of more severe illness-they are more likely to recognize the severity of health problems, creating a strong need for health information. This need motivates them to proactively utilize various resources (e.g., digital platforms, healthcare providers) to seek information related to disease prevention, treatment, and rehabilitation in order to improve their health. Family members possessing medical literacy are capable of converting professional medical knowledge into practical health guidance, thus delivering tailored recommendations to their relatives (51). This process not only improves the health literacy of family members through evidence-based education, but also effectively promotes the adoption of positive health behaviors. Furthermore, social support was a key factor in this study. The data showed a positive correlation between the level of social support and rural residents’ participation in health communication behaviors: the higher social support, the higher the motivation to participate in health promoting activities. This indicates that a supportive and caring environment strengthens individuals’ confidence and motivation to engage in health communication initiatives (47).

In terms of the environmental level, the frequency of village health communication was the only significant factor, which highlights the key role of community-level health information supply in rural areas. This result suggests that rural residents’ health communication behaviors are highly dependent on the regular, accessible, and sustainable health promotion activities organized by village committees and community health institutions (52). This continuous information output not only enhances villagers’ awareness of health issues, but also facilitates their participation in health communication activities through interpersonal interactions and knowledge sharing in social networks. From a policy implication perspective, this finding underscores the importance of strengthening the rural grassroots health communication system (53). Policy makers should prioritize increasing the frequency and quality of village-level health education activities, establishing regular health information dissemination mechanisms, and equipping village clinics with standardized health communication materials. Moreover, integrating health communication into the basic public health services for rural residents could further improve the accessibility and effectiveness of health information, thereby promoting more active health communication behaviors at the population level.

### Social support constitutes the central factor within the network

The multivariable regression model only identifies independent predictive effects of each factor, whereas network analysis is able to capture potential interactions and interdependencies across ecological levels that may not be detected in regression (54). For instance, social support may moderate or mediate the effects of individual factors and environmental factors on health communication behaviors. Such cross-level interactions are consistent with the core logic of the social-ecological model, which emphasizes that health behaviors arise from dynamic interactions across multiple levels rather than simple additive effects. Specifically, social support occupies a central position in this network, exhibiting the highest level of strength, betweenness and closeness, making it the most critical factor. Interventions on this central factor can mitigate the effects of inhibitory factors while activating the links between facilitators (55). Simultaneously, social support serves as a “bridge” between individual-level factors (e.g., perceived usefulness, media engagement, occupation, and monthly household income) and other system components. For example, higher-income households have access to richer social resources and thus a broader social support networks. Individuals with high media involvement and perceived ease of use scores actively or subconsciously assess the accessibility, professionalism, and usefulness of health information. Villagers will be willing to share and engage with health content when this information is easily accessible, professionally sourced and practical (49). At the interpersonal level, emergencies such as family illness are closely related to social support networks. For example, chronic disease experiences may prompt individuals to seek emotional and instrumental support, thereby increasing their need for health information (50). Although individual and interpersonal factors dominated the network’s centrality indices, the significant predictive role of health communication frequency from village committees highlights the important impact of the local community environment. In rural China, where informal social ties are strong, village committees function as authoritative and trusted sources of public health messaging (56). Additionally, age, gender, and family medical history showed significant interconnections: age emerged as a critical determinant of health needs, which was particularly evident in the older adults requiring chronic disease management (57). From a systems perspective, the health communication behavior of rural residents is a dynamic process shaped by the interactions of internal and external factors. Changes in the level of health communication activity levels reflect the stage-by-stage results of these complex interactions.

It is noteworthy that the environmental level in this study comprised fewer variables compared to the individual and interpersonal levels. While the network analysis included only predictors that were statistically significant in the regression models, this disparity in variable count may inherently bias centrality metrics toward factors with greater representation in the network. Consequently, the apparent dominance of individual and interpersonal factors in the network centrality indices should be interpreted with caution. Future studies should incorporate a more comprehensive array of environmental indicators (e.g., community infrastructure) to achieve a balanced representation across ecological levels.

## Conclusion

Based on the social-ecological model, this study integrates multilevel regression analysis and network analysis to clarify the complex correlational mechanisms among various factors influencing health communication behaviors among rural residents. The findings suggest that the overall level of health communication behaviors of rural residents remains low, and that these behaviors are shaped by multi-level and multi-factorial interactions. Individual factors such as age, gender, income, media involvement, and perceived ease of use; interpersonal factors including types of chronic disease in the family, family medical background, and social support; and the environmental factor of village health communication frequency all played significant roles. Notably, network analysis revealed that social support was the core hub of the system, exhibiting the highest strength, betweenness and closeness. This indicates that social support is not merely a predictive factor but also a critical bridging mechanism: it closely links individual attributes with interpersonal dynamics and environmental factors. Other key determinants include types of chronic disease in the family, perceived ease of use, media involvement, and occupation. These findings have important implications for the design of interventions. Effective strategies should not be limited to single-dimensional approaches, but rather adopt a coordinated, multi-pronged framework. Priority should be given to strengthening social support systems, particularly at the family and community levels. Policy initiatives should aim to empower village committees to act as catalysts for mobilizing social support, while addressing individual-level barriers such as digital literacy and perceived ease of use, thereby maximizing the reach and impact of health communication programs in rural areas.

### Limitations

Several limitations of the study are noteworthy. First, the cross-sectional design does not allow us to infer causality, so a longitudinally designed study is needed to validate these findings. Second, individual, interpersonal, and environmental factors are all critical, but the number of environmental variables was relatively small, which may have affected the weight distribution of nodes in the network analysis. Future studies should include more environmental indicators to obtain a more balanced network structure. Third, non-significant variables were not included, which may ignore some potential indirect effects. Future studies may use full-variable network models for sensitivity comparison. Fourthly, the study was based on a sample from Ningxia, which limits the generalizability of the findings to other regions. Subsequent studies should expand the sampling area to include more representative rural areas through field surveys to ensure comprehensive data collection in order to expand the applicability of the study.

## Data Availability

The raw data supporting the conclusions of this article will be made available by the authors, without undue reservation.
